# Supplementary material for: Hypertrophic cardiomyopathy disease results from disparate impairments of cardiac myosin function and auto-inhibition
Source: Nat Commun. 2018 Oct 1;9:4019. doi: 10.1038/s41467-018-06191-4 (PMC6167380; doi:10.1038/s41467-018-06191-4)
Supplement: Supplementary file 1 — Supplementary Information [file 41467_2018_6191_MOESM1_ESM.pdf]

Hypertrophic cardiomyopathy disease results from disparate impairments of cardiac myosin function and auto-inhibition

Robert-Paganin et al., 2018

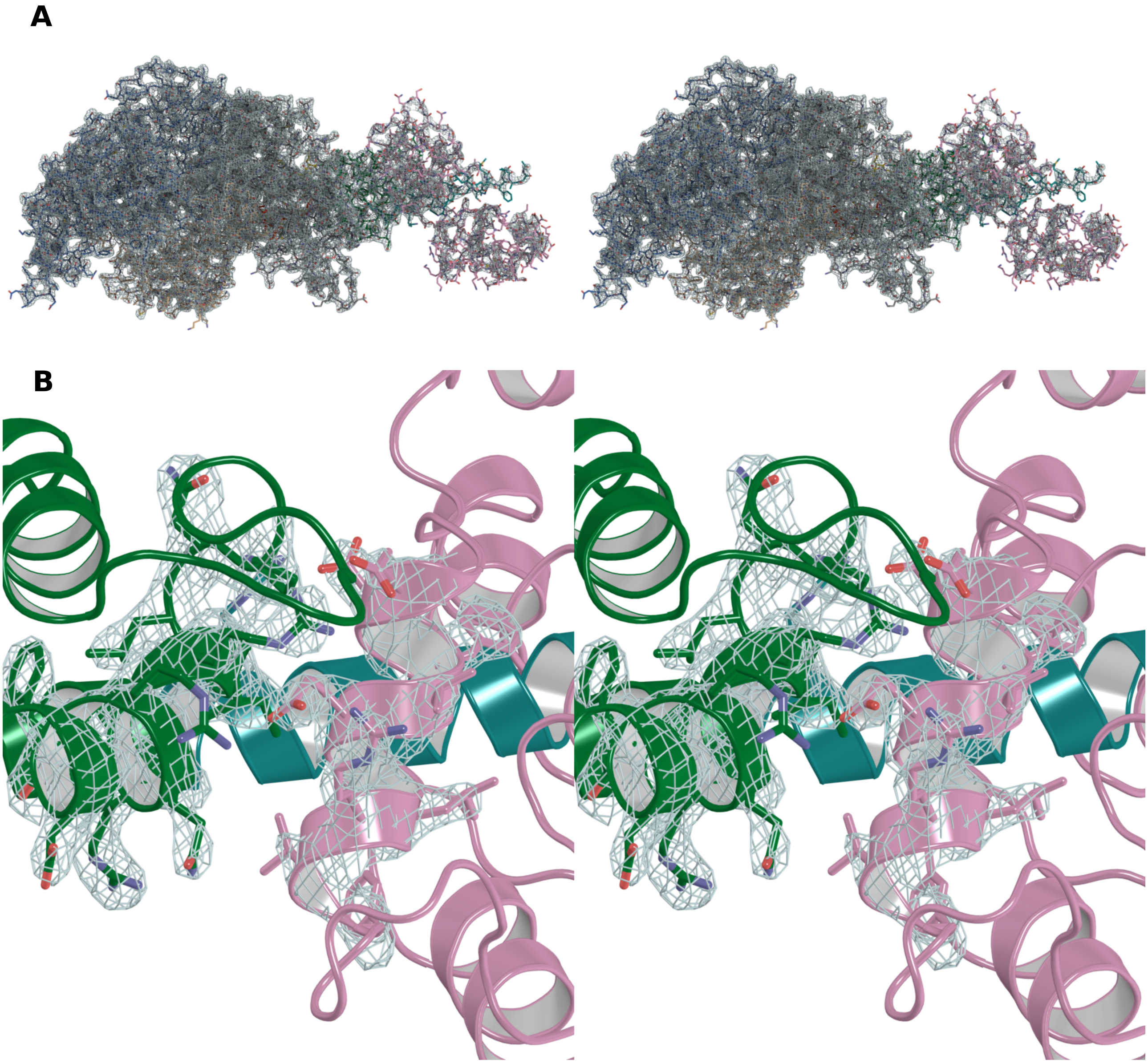

Supplementary Figure 1: Electron density map of the post-rigor S1 (PR-S1) crystal structure and the ELC/converter interface

(A) Stereo view of the electron density map of the crystal structure of the β-cardiac myosin S1 fragment in the post-rigor state (PR-S1). The electron density map in pale cyan corresponds to a 2Fo-Fc map with a contour of 1.0 σ. The different subdomains of the model (stick representation) are colored: N-terminus (grey); U50 (marine blue); L50 (wheat); relay (yellow); SH1 helix (red); converter (green); lever arm (cyan); ELC (light pink). **(B)** Stereo view of the electron density map (2Fo-Fc; contour of 1.0 σ) for the converter/ELC interface. Residues involved in the interface are well defined in electron density and the side chains can be clearly identified. The ELC backbone is clearly defined as well as the side chains of the E helix that interacts with the converter.

A

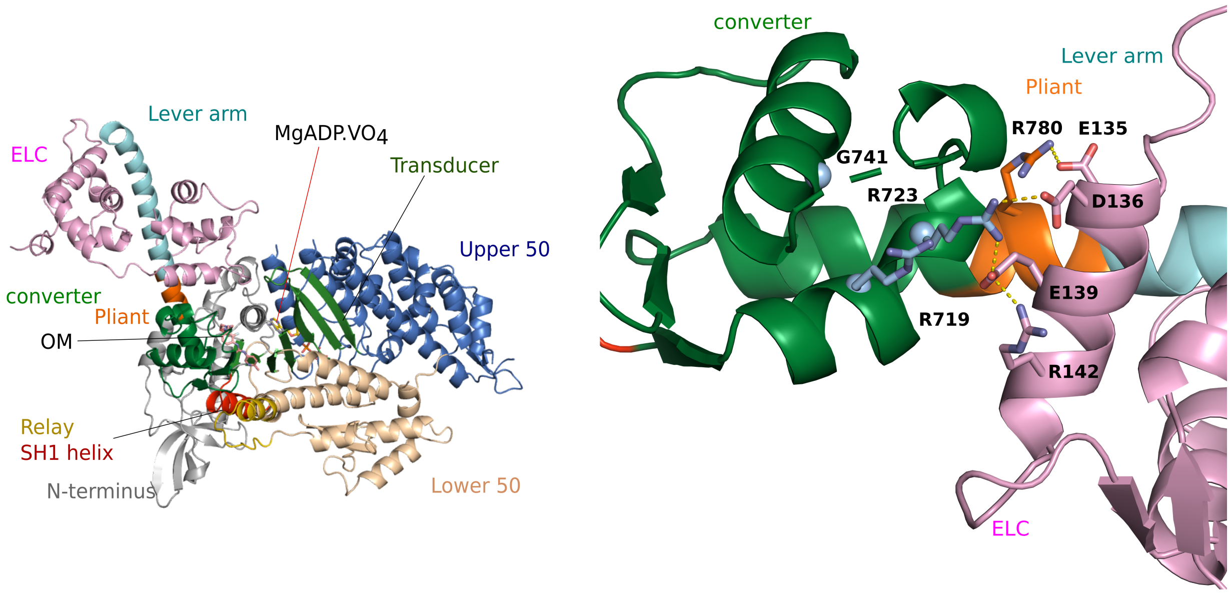

B

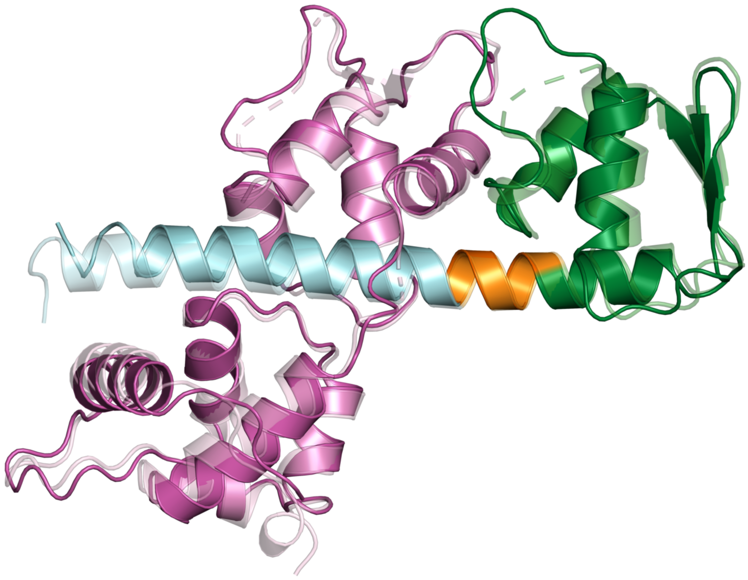

Supplementary Figure 2: The ELC/converter interface and the lever arm of the β-cardiac myosin are conserved in structure in the post-rigor (PR) and in the pre-powerstroke (PPS) states

(A) On the left, X-ray structure of β-cardiac myosin S1 bound to the activator omecamtiv mecarbil (OM) in the pre-powerstroke state (PDB code: 5N69). On the right, interface between the converter and the ELC as found in the PPS-state. **(B)** Cartoon representation of the lever arm region (converter, pliant, IQ motif and ELC) of β-cardiac myosin in the PR state (same color code as in Fig. 2A and 2B) superimposed with that found in the PPS state structure (translucid) (PDB code: 5N69). Both structures superimpose on this region with a root mean square deviation (RMSD) of 0.982 Å.

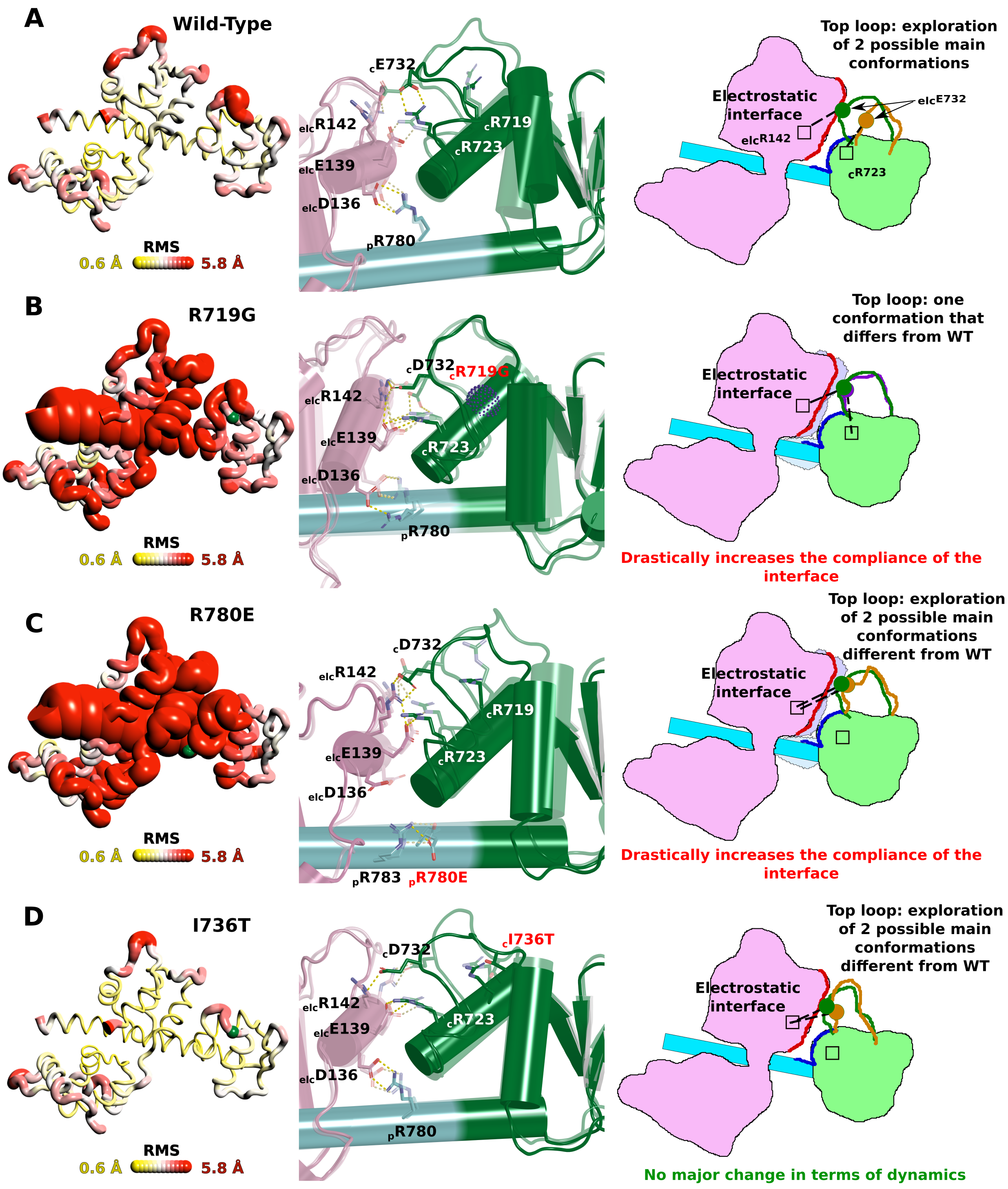

Supplementary Figure 3: Dynamics of the Converter/ELC interface

Schematic representation of the results from the molecular dynamics simulations for the wild-type (WT), two proof-of-concept mutations (R719A and R780E) as well as one HCM (I736T) mutant that have been analyzed in silico. On the left, a Putty representation is displayed for a β-cardiac myosin construct that includes the residues 701-806 and the ELC. Root mean square (RMS) fluctuations during 30 ns simulations are represented with a RMS scale ranging from 0.6 Å (in yellow) to 5.8 Å (in red). Structures represented here correspond to the most populated structural state. In each structure, the position of the residue mutated is labeled in red. On the center, a schematic representation of the region containing the converter, the essential light chain (ELC) and the lever arm is displayed. The different populations of the top loop allowed by the dynamics of this region are drawn and the nature of the interactions between the converter and the ELC is also represented. In each case a state is represented opaque and the others are in transparency in order to best compare the different populations. On the right, the interface between the ELC, the converter and the pliant region is represented. The different positions (positions are colored differently) schematized on the right are represented on the structure with the position of all key-residues that maintain the interface and its plasticity. On the center and on the left, the myosin subdomains are colored differently: the converter in green; the IQ region in cyan and the ELC in light pink. **(A)** WT, **(B)** R719G, **(C)** R780E, **(D)** I736T.

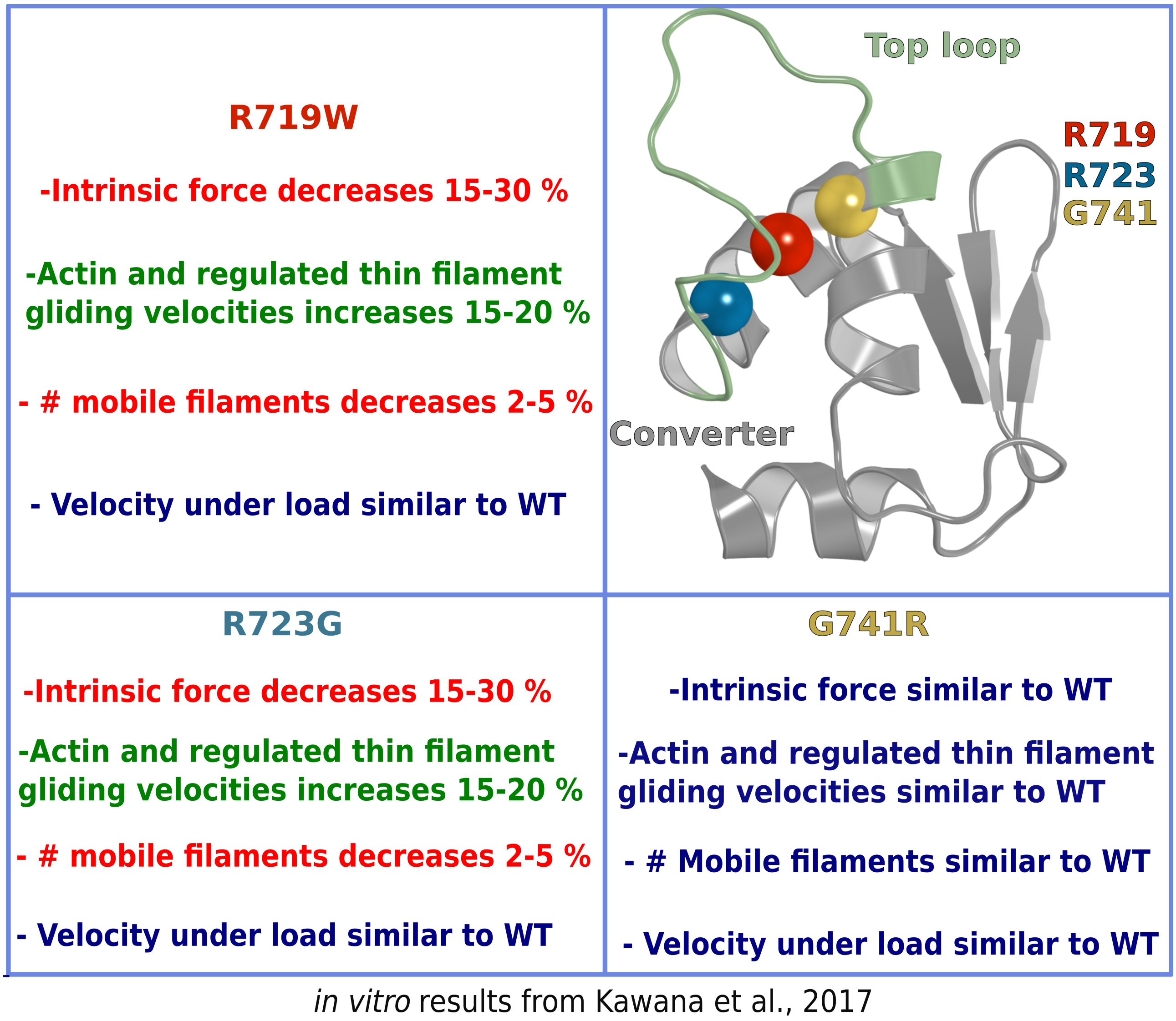

Supplementary Figure 4: Consequences of HCM mutations in the converter on the biomechanical properties of β-cardiac myosin as reported from the Kawana et al. studies performed in the Spudich laboratory.
Summary of the consequences of the mutations R719W, R723G and G741R compared to WT for the biomechanical properties of the cardiac myosin motor[83]. On the top, right, the three mutations are located (colored spheres) on a cartoon representation of the β-cardiac myosin converter.

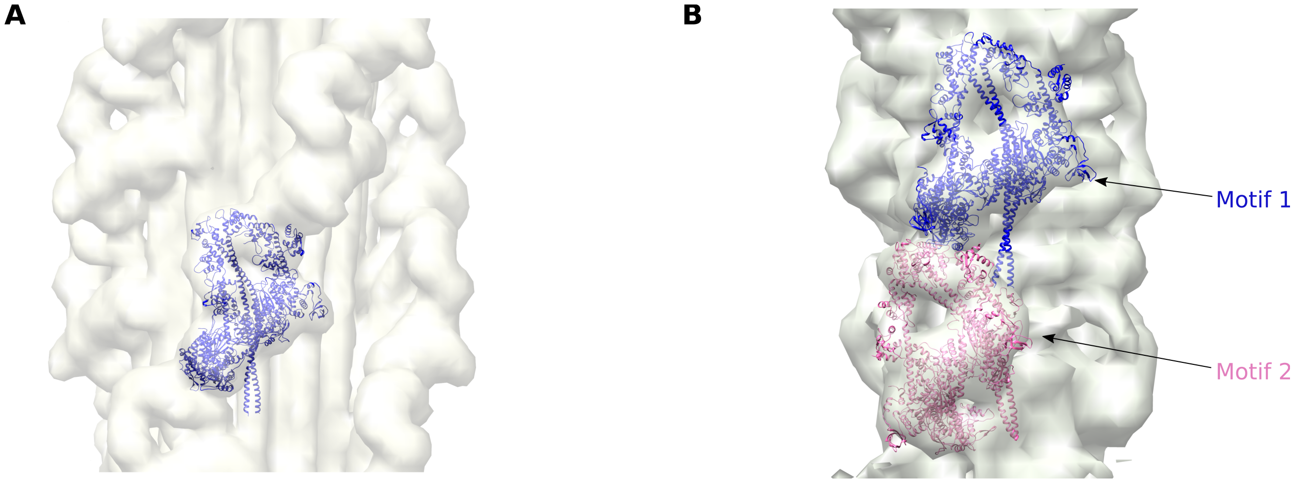

Supplementary Figure 5: Sequestered state model and its fit in the electron density map
(A) Fit of our model in the electron density of tarantula muscle filament (EMD code: EMD-1950). **(B)** Reconstitution of the cardiac filament and sequestered state. Fit of our model in the human cardiac muscle filament electron density map obtained from negative stained electron microscopy (EM) data (EMDB code EMD-2240). Since the geometry in cardiac filament is not purely helical[98], two motifs have been fitted in the filament, each motif is slightly rotated compared to the other.

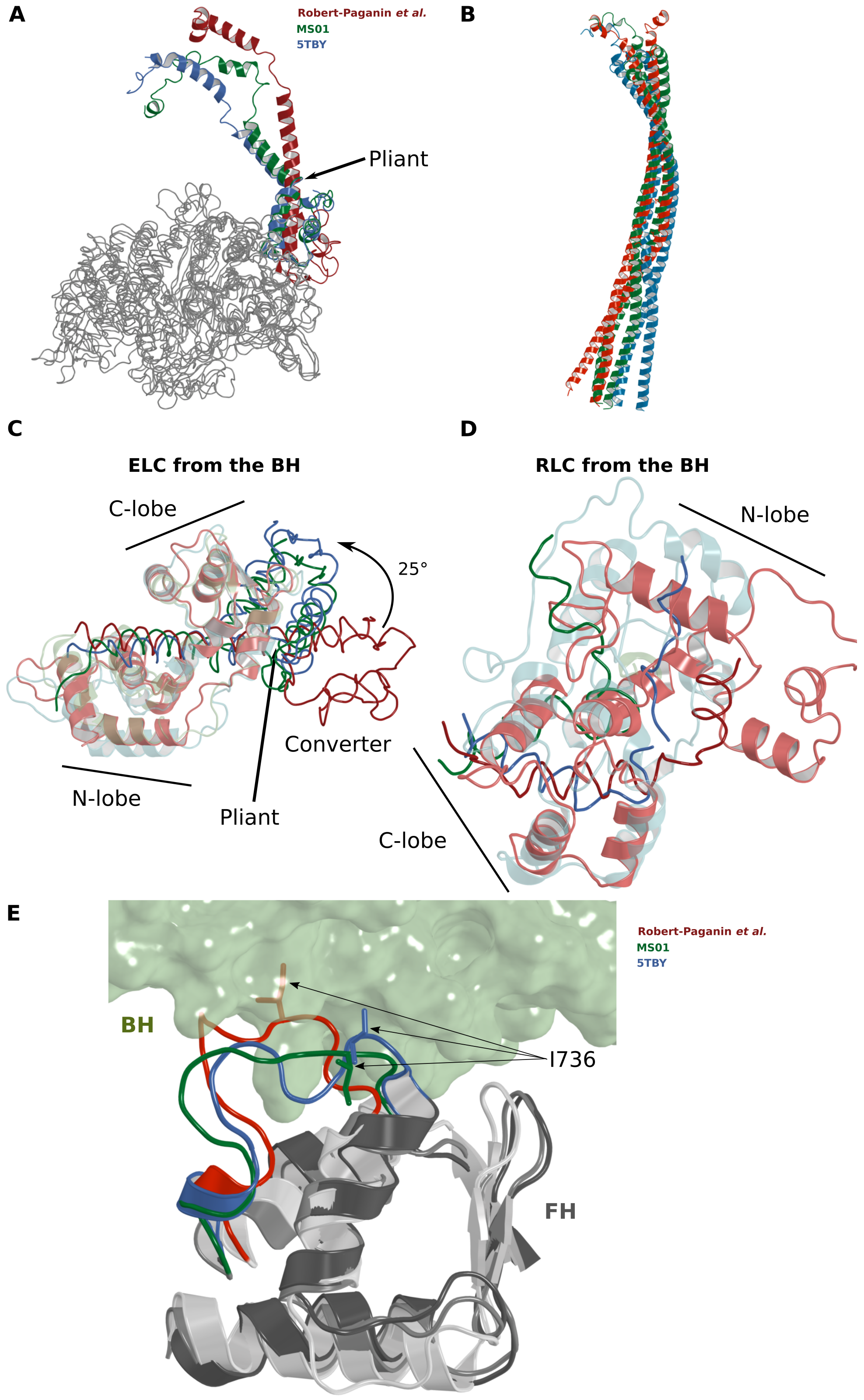

Supplementary Figure 6: Comparison of sequestered state models

Comparison of the sequestered model we propose (red) with the two previously released models: MS01 (green) from Jim Spudich's lab[37] and 5TBY (blue) from Raul Padron's lab[8]. All the models have been superimposed on the N-ter region (1-171) of the blocked head (BH) of our model. **(A)** Comparison of the converter/lever arm region that is displayed in cartoon models, **(B)** Comparison of the S2 region. **(C)** Comparison of the converter/ELC region. **(D)** Comparison of the RLC region, RLC are aligned on the C-lobe. **(E)** Comparison of the interface between the FH-converter (cartoon) and the BH (transparent surface) in the model we present here (converter in white, top loop in red), the MS01 model (converter in light grey, top loop in green), and the 5TBY model (converter in dark grey, top loop in blue). For each structure, the side chain of Ile 736 is represented, showing that only in our model is this residue modeled in the interface between both heads.

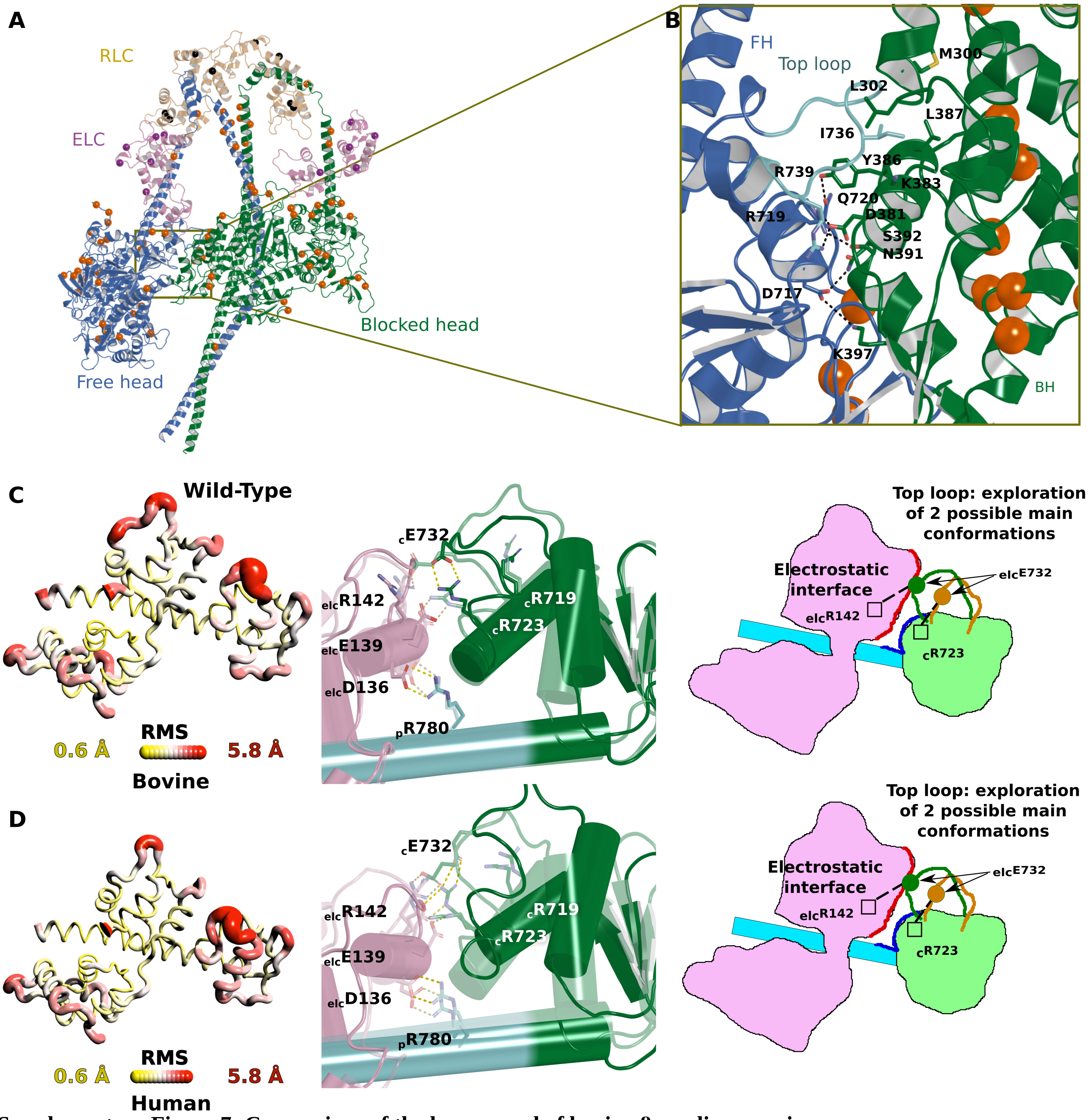

Supplementary Figure 7: Comparison of the human and of bovine β-cardiac myosins

Cartoon representation of the minimized model of the sequestered state of human β-cardiac myosin. Polymorphism are represented as balls colored in orange, purple and black for the heavy chain, the essential light chain (ELC) and the regulatory light chain (RLC) respectively. **(B)** Region of interaction between the blocked head (BH) and the free head (FH), the top loop is colored in cyan. The interfaces are identical in the human and bovine models. **(C)** and **(D)** represent results from molecular dynamics on the lever arm of the bovine β-cardiac myosin **(C)** and of the human β-cardiac myosin **(D)**. On the left, a putty representation is displayed for a β-cardiac myosin construct that includes the residues 701-806 and the ELC. RMS fluctuations during 30 ns simulations are represented with a RMS scale ranging from 0.6 Å (in yellow) to 5.8 Å (in red). Structures represented here correspond to the most populated structural states. On the center, the interface between the ELC, the converter and the pliant region is represented. Different conformations are colored differently on the structure with the position of all key-residues that maintain the interface and its plasticity. A state is represented opaque and the others are in transparency in order to best compare the different populations. On the right, a schematic representation of the region containing the converter, the ELC and the lever arm is displayed. The different populations of the top loop allowed by the dynamics of this region are drawn and the nature of the interactions between the converter and the ELC is also represented. On the center and on the left, the myosin subdomains are colored differently: the converter in green; the IQ region in cyan and the ELC in light pink.

Supplementary Table 1: Classification of a set of 178 HCM mutations depending on their effects.

Residue directly involved in IHM interactions; Indirect effect on IHM interactions; **early onset HCM**

Sequestered state destabilized by direct effect on IHM without impairment in motor activity

Mesa + PPS stabilization	R169G	E170K							
Not mesa – surface	K351E	E379K	**D382Y**	K383N	Y386H	L390V			
Mesa	D394E		K615N	K615Q	T660N	R663H	R663S		
Possible Allosteric effect	S384P	A385V							
Relay (mesa)	E499K								
Converter IHM interface	**G716R**	G716A	D717G	A728V	I730M	I730N			
	P731L	P731S	G733E	Q734E	Q734P	I736M	**I736T**	K740N	E743D

Sequestered state destabilized due to effect on IHM contacts – Motor function altered

Transducer	R249Q	R249N	**H251N**	K450E	K450T	**R453C**	R453H	R453S
Actin surface and IHM	F312C	**A355T**	**R403Q**	R403L	R403W	R403G	V404M	V404L
	G407V	N408K	N437D	I530V	M539L	R652G		
Converter	R719Q	R719W	R719P	R723C	R723G	**G741R**	G741W	A742E

Sequestered state destabilized due to effect on PPS stability or off state formation – Motor function altered

A- Mutations impairing function and predicted to destabilize the PPS conformation and thus the off state

Nter/conv in PPS state	**K146N**	S148I							
Loop1/transducer	R204H	K207Q	P211L	D218Y	N219E				
Switch I	T235N	**D239N**							
Switch II	D461E	N471S	N479S						
Relay	M493L	E497D	N498R	E500A	Y501C	I506T			
	I511F	I511T	F513C	M515R	M515V	M515T			
	D516E	L517M							
Converter/Relay/SH1 helix	N696C	V698A	I702V	**P710R**	R712L	K762R	F764Y	K766Q	G768R

B- Mutations impairing the compliance required for the formation of the sequestered state

Converter /pliant region	D778E	D778G	D778N	D778V	
Lever arm compliance	L781P	L781M			alteration of the pliant region and thus the formation of the off-state
	S782N	S782R			
	R783C	R783H			

C- Mutations impairing function and that <u>may</u> also destabilize the PPS conformation and thus the sequestered state

Transducer	A196T	A199T	Q222K	F247L	**G256E**	R671C	M690T
Switch I	N232S	F244L	V320M				

Protein stability likely altered as well as motor function – possible effect on the off state if the PPS stability is altered

Near active site	Y115H	T124I	T135I	N187K	R190T	E328G
Nter	R143G	R143Q	Y162C	T188N		
Nter near joints / active site	T177I	V186L				
Transducer	I263M	I263T				
U50	I303M	P307H	G425R	A428V	R434T	M435T
	M439T	V440M	V440A	R442C	I443T	N444S
L50	E483K	G584R	G584S	M659I		
Converter	V763G	**V763M**	E774V			

Motor function/stability mildly altered – likely no major effect on sequestered state

Nter linker/ subdomain	S4L	A26V	V39M	F46I	V52M	V59I	I87T	F95I	A100T	R694C	R694H
Surface U50 (not part of mesa)	Y308C	D309N									
U50 core	L227V	M349T									

Motor function altered – without predicted effect on IHM

Mutations for which no effect on PPS is predicted => no effect on sequestered state

Powerstroke altered	A426T (transition slower after Pi release)					A430E		
Actin surface	V406M	V411I	I524L	K542R	G571R	H576R	D587V	**V606M**
L50 near connectors	A583V							
Strut	N602S	E603K						

Supplementary References:

1. Homburger, J. R. *et al.* Multidimensional structure-function relationships in human beta-cardiac myosin from population-scale genetic variation. *Proc Natl Acad Sci U S A* **113,** 6701–6706 (2016).

2. Song, L. *et al.* Mutations profile in Chinese patients with hypertrophic cardiomyopathy. *Clin. Chim. Acta* **351,** 209–216 (2005).

3. Richard, P. Hypertrophic Cardiomyopathy: Distribution of Disease Genes, Spectrum of Mutations, and Implications for a Molecular Diagnosis Strategy. *Circulation* **107,** 2227–2232 (2003).

4. Wang, J. *et al.* Malignant effects of multiple rare variants in sarcomere genes on the prognosis of patients with hypertrophic cardiomyopathy. *Eur. J. Heart Fail.* **16,** 950–957 (2014).

5. Bos, J. M. *et al.* Characterization of a Phenotype-Based Genetic Test Prediction Score for Unrelated Patients With Hypertrophic Cardiomyopathy. *Mayo Clin. Proc.* **89,** 727–737 (2014).

6. Gasteiger, E. *et al.* ExPASy: The proteomics server for in-depth protein knowledge and analysis. *Nucleic Acids Res.* **31,** 3784–3788 (2003).

7. Van Driest, S. L. *et al.* Comprehensive Analysis of the Beta-Myosin Heavy Chain Gene in 389 Unrelated Patients With Hypertrophic Cardiomyopathy. *J. Am. Coll. Cardiol.* **44,** 602–610 (2004).

8. Alamo, L. *et al.* Effects of myosin variants on interacting-heads motif explain distinct hypertrophic and dilated cardiomyopathy phenotypes. *Elife* **6,** 2386–2390 (2017).

9. Rayment, I., Holden, H. M., Sellers, J. R., Fananapazir, L. & Epstein, N. D. Structural interpretation of the mutations in the beta-cardiac myosin that have been implicated in familial hypertrophic cardiomyopathy. *Proc. Natl. Acad. Sci. U. S. A.* **92,** 3864–3868 (1995).

10. Mohiddin, S. A. *et al.* Utility of genetic screening in hypertrophic cardiomyopathy: prevalence and significance of novel and double (homozygous and heterozygous) beta-myosin mutations. *Genet. Test.* **7,** 21–27 (2003).

11. Ingles, J. Compound and double mutations in patients with hypertrophic cardiomyopathy: implications for genetic testing and counselling. *J. Med. Genet.* **42,** e59–e59 (2005).

12. Morita, H. *et al.* Shared Genetic Causes of Cardiac Hypertrophy in Children and Adults. *N. Engl. J. Med.* **358,** 1899–1908 (2008).

13. Trivedi, D. V., Adhikari, A. S., Sarkar, S. S., Ruppel, K. M. & Spudich, J. A. Hypertrophic cardiomyopathy and the myosin mesa: viewing an old disease in a new light. *Biophys. Rev.* **10,** 27–48 (2018).

14. Kassem, H. S. *et al.* Early Results of Sarcomeric Gene Screening from the Egyptian National BA-HCM Program. *J. Cardiovasc. Transl. Res.* **6,** 65–80 (2013).

15. Millat, G. *et al.* Prevalence and spectrum of mutations in a cohort of 192 unrelated patients with hypertrophic cardiomyopathy. *Eur. J. Med. Genet.* **53,** 261–267 (2010).

16. Bundgaard, H. *et al.* Familial Hypertrophic Cardiomyopathy Associated with a Novel Missense

Mutation Affecting the ATP-binding Region of the Cardiac Beta-myosin Heavy Chain. *J. Mol. Cell. Cardiol.* **31,** 745–750 (1999).

17. Havndrup, O. *et al.* Outcome of clinical versus genetic family screening in hypertrophic cardiomyopathy with focus on cardiac beta-myosin gene mutations. *Cardiovasc. Res.* **57,** 347–357 (2003).

18. Woo, A. *et al.* Mutations of the beta myosin heavy chain gene in hypertrophic cardiomyopathy: critical functional sites determine prognosis. *Heart* **89,** 1179–1185 (2003).

19. Perrot, A. *et al.* Prevalence of cardiac beta-myosin heavy chain gene mutations in patients with hypertrophic cardiomyopathy. *J. Mol. Med.* **83,** 468–477 (2005).

20. Pylypenko, O. *et al.* Myosin VI deafness mutation prevents the initiation of processive runs on actin. *Proc. Natl. Acad. Sci.* **112,** E1201–E1209 (2015).

21. Yu, B. *et al.* Denaturing high performance liquid chromatography: high throughput mutation screening in familial hypertrophic cardiomyopathy and SNP genotyping in motor neurone disease. *J. Clin. Pathol.* **58,** 479–485 (2005).

22. Kaski, J. P. *et al.* Prevalence of sarcomere protein gene mutations in preadolescent children with hypertrophic cardiomyopathy. *Circ. Cardiovasc. Genet.* **2,** 436–441 (2009).

23. Adhikari, A. S. *et al.* Early-Onset Hypertrophic Cardiomyopathy Mutations Significantly Increase the Velocity, Force, and Actin-Activated ATPase Activity of Human beta-Cardiac Myosin. *Cell Rep* **17,** 2857–2864 (2016).

24. Watkins, H. *et al.* Characteristics and Prognostic Implications of Myosin Missense Mutations in Familial Hypertrophic Cardiomyopathy. *N. Engl. J. Med.* **326,** 1108–1114 (1992).

25. Arbustini, E. *et al.* Coexistence of mitochondrial DNA and beta myosin heavy chain mutations in hypertrophic cardiomyopathy with late congestive heart failure. *Heart* **80,** 548–558 (1998).

26. Greber-Platzer, S. *et al.* Beta-myosin Heavy Chain Gene Mutations and Hypertrophic Cardiomyopathy in Austrian Children. *J. Mol. Cell. Cardiol.* **33,** 141–148 (2001).

27. Waldmuller, S., Freund, P., Mauch, S., Toder, R. & Vosberg, H.-P. Low-density DNA microarrays are versatile tools to screen for known mutations in hypertrophic cardiomyopathy. *Hum. Mutat.* **19,** 560–569 (2002).

28. Fananapazir, L., Dalakas, M. C., Cyran, F., Cohn, G. & Epstein, N. D. Missense mutations in the beta-myosin heavy-chain gene cause central core disease in hypertrophic cardiomyopathy. *Proc. Natl. Acad. Sci. U. S. A.* **90,** 3993–3997 (1993).

29. Tesson, F. *et al.* Genotype-phenotype analysis in four families with mutations in beta-myosin heavy chain gene responsible for familial hypertrophic cardiomyopathy. *Hum. Mutat.* **12,** 385–392 (1998).

30. Jeschke, B. *et al.* A high risk phenotype of hypertrophic cardiomyopathy associated with a compound genotype of two mutated beta-myosin heavy chain genes. *Hum. Genet.* **102,** 299–304 (1998).

31. Kuang, S. Q. *et al.* Identification of a novel missense mutation in the cardiac beta-myosin heavy chain gene in a Chinese patient with sporadic hypertrophic cardiomyopathy. *J. Mol. Cell. Cardiol.* **28,** 1879–1883 (1996).

32. Havndrup, O. *et al.* A novel missense mutation, Leu390Val, in the cardiac beta-myosin heavy chain associated with pronounced septal hypertrophy in two families with hypertrophic cardiomyopathy. *Scand. Cardiovasc. J.* **34,** 558–563 (2000).

33. Geisterfer-Lowrance, A. A. *et al.* A molecular basis for familial hypertrophic cardiomyopathy: a beta cardiac myosin heavy chain gene missense mutation. *Cell* **62,** 999–1006 (1990).

34. Epstein, N. D., Cohn, G. M., Cyran, F. & Fananapazir, L. Differences in clinical expression of hypertrophic cardiomyopathy associated with two distinct mutations in the beta-myosin heavy chain gene. A 908Leu----Val mutation and a 403Arg----Gln mutation. *Circulation* **86,** 345–352 (1992).

35. Watkins, H. *et al.* Independent origin of identical beta cardiac myosin heavy-chain mutations in hypertrophic cardiomyopathy. *Am. J. Hum. Genet.* **53,** 1180–1185 (1993).

36. Cuda, G., Fananapazir, L., Zhu, W. S., Sellers, J. R. & Epstein, N. D. Skeletal muscle expression and abnormal function of beta-myosin in hypertrophic cardiomyopathy. *J. Clin. Invest.* **91,** 2861–2865 (1993).

37. Nag, S. *et al.* The myosin mesa and the basis of hypercontractility caused by hypertrophic cardiomyopathy mutations. *Nat. Struct. Mol. Biol.* **24,** 525–533 (2017).

38. Sweeney, H. L., Straceski, A. J., Leinwand, L. A., Tikunov, B. A. & Faust, L. Heterologous expression of a cardiomyopathic myosin that is defective in its actin interaction. *J. Biol. Chem.* **269,** 1603–1605 (1994).

39. Roopnarine, O. & Leinwand, L. A. Functional Analysis of Myosin Mutations That Cause Familial Hypertrophic Cardiomyopathy. *Biophys. J.* **75,** 3023–3030 (1998).

40. Blanchard, E., Seidman, C., Seidman, J. G., LeWinter, M. & Maughan, D. Altered crossbridge kinetics in the alphaMHC403/+ mouse model of familial hypertrophic cardiomyopathy. *Circ. Res.* **84,** 475–483 (1999).

41. Lankford, E. B., Epstein, N. D., Fananapazir, L. & Sweeney, H. L. Abnormal contractile properties of muscle fibers expressing beta-myosin heavy chain gene mutations in patients with hypertrophic cardiomyopathy. *J. Clin. Invest.* **95,** 1409–1414 (1995).

42. Tyska, M. J. *et al.* Single-molecule mechanics of R403Q cardiac myosin isolated from the mouse model of familial hypertrophic cardiomyopathy. *Circ. Res.* **86,** 737–744 (2000).

43. Yamashita, H., Tyska, M. J., Warshaw, D. M., Lowey, S. & Trybus, K. M. Functional consequences of mutations in the smooth muscle myosin heavy chain at sites implicated in familial hypertrophic cardiomyopathy. *J. Biol. Chem.* **275,** 28045–28052 (2000).

44. Debold, E. P. *et al.* Hypertrophic and dilated cardiomyopathy mutations differentially affect the molecular force generation of mouse α-cardiac myosin in the laser trap assay. *Am. J. Physiol. Circ. Physiol.* **293,** H284–H291 (2007).

45. Volkmann, N. *et al.* The R403Q Myosin Mutation Implicated in Familial Hypertrophic Cardiomyopathy Causes Disorder at the Actomyosin Interface. *PLoS One* **2,** e1123 (2007).

46. Malinchik, S., Cuda, G., Podolsky, R. J. & Horowits, R. Isometric tension and mutant myosin heavy chain content in single skeletal myofibers from hypertrophic cardiomyopathy patients. *J. Mol. Cell. Cardiol.* **29,** 667–676 (1997).

47. Palmiter, K. A. *et al.* R403Q and L908V mutant beta-cardiac myosin from patients with familial hypertrophic cardiomyopathy exhibit enhanced mechanical performance at the single molecule level. *J. Muscle Res. Cell Motil.* **21,** 609–620 (2000).

48. Lowey, S. *et al.* Functional effects of the hypertrophic cardiomyopathy R403Q mutation are different in an alpha- or beta-myosin heavy chain backbone. *J. Biol. Chem.* **283,** 20579–20589 (2008).

49. Belus, A. *et al.* The familial hypertrophic cardiomyopathy-associated myosin mutation R403Q accelerates tension generation and relaxation of human cardiac myofibrils. *J. Physiol.* **586,** 3639–3644 (2008).

50. Lowey, S., Bretton, V., Gulick, J., Robbins, J. & Trybus, K. M. Transgenic mouse alpha- and beta-cardiac myosins containing the R403Q mutation show isoform-dependent transient kinetic differences. *J. Biol. Chem.* **288,** 14780–14787 (2013).

51. Sata, M. & Ikebe, M. Functional analysis of the mutations in the human cardiac beta-myosin that are responsible for familial hypertrophic cardiomyopathy. Implication for the clinical outcome. *J. Clin. Invest.* **98,** 2866–2873 (1996).

52. Nag, S. *et al.* Contractility parameters of human -cardiac myosin with the hypertrophic cardiomyopathy mutation R403Q show loss of motor function. *Sci. Adv.* **1,** e1500511–e1500511 (2015).

53. Dausse, E. *et al.* Familial hypertrophic cardiomyopathy. Microsatellite haplotyping and identification of a hot spot for mutations in the beta-myosin heavy chain gene. *J. Clin. Invest.* **92,** 2807–2813 (1993).

54. Moolman, J. C., Brink, P. A. & Corfield, V. A. Identification of a new missense mutation at Arg403, a CpG mutation hotspot, in exon 13 of the beta-myosin heavy chain gene in hypertrophic cardiomyopathy. *Hum. Mol. Genet.* **2,** 1731–1732 (1993).

55. Moolman-Smook, J. C., De Lange, W. J., Bruwer, E. C., Brink, P. A. & Corfield, V. A. The origins of hypertrophic cardiomyopathy-causing mutations in two South African subpopulations: a unique profile of both independent and founder events. *Am. J. Hum. Genet.* **65,** 1308–1320 (1999).

56. Erdmann, J. *et al.* Mutation spectrum in a large cohort of unrelated consecutive patients with hypertrophic cardiomyopathy. *Clin. Genet.* **64,** 339–49 (2003).

57. Keller, D. I. *et al.* Human homozygous R403W mutant cardiac myosin presents disproportionate enhancement of mechanical and enzymatic properties. *J. Mol. Cell. Cardiol.* **36,** 355–362 (2004).

58. Llinas, P. *et al.* How Actin Initiates the Motor Activity of Myosin. *Dev. Cell* **33,** 401–412 (2015).

59. Morner, S. *et al.* Identification of the genotypes causing hypertrophic cardiomyopathy in northern Sweden. *J. Mol. Cell. Cardiol.* **35,** 841–849 (2003).

60. Roncarati, R. *et al.* Unexpectedly low mutation rates in beta-myosin heavy chain and cardiac myosin binding protein genes in Italian patients with hypertrophic cardiomyopathy. *J. Cell. Physiol.* **226,** 2894–2900 (2011).

61. Ko, Y. L. *et al.* Malignant familial hypertrophic cardiomyopathy in a family with a 453Arg-->Cys mutation in the beta-myosin heavy chain gene: coexistence of sudden death and end-stage

heart failure. *Hum. Genet.* **97,** 585–590 (1996).

62. Nanni, L. *et al.* Hypertrophic cardiomyopathy: two homozygous cases with 'typical' hypertrophic cardiomyopathy and three new mutations in cases with progression to dilated cardiomyopathy. *Biochem. Biophys. Res. Commun.* **309,** 391–8 (2003).

63. Sommese, R. F. *et al.* Molecular consequences of the R453C hypertrophic cardiomyopathy mutation on human -cardiac myosin motor function. *Proc. Natl. Acad. Sci.* **110,** 12607–12612 (2013).

64. Bloemink, M. *et al.* The Hypertrophic Cardiomyopathy Myosin Mutation R453C Alters ATP Binding and Hydrolysis of Human Cardiac β-Myosin. *J. Biol. Chem.* **289,** 5158–5167 (2014).

65. Frazier, A. *et al.* Familial hypertrophic cardiomyopathy associated with cardiac beta-myosin heavy chain and troponin I mutations. *Pediatr. Cardiol.* **29,** 846–850 (2008).

66. Kubo, T. *et al.* Prevalence, Clinical Significance, and Genetic Basis of Hypertrophic Cardiomyopathy With Restrictive Phenotype. *J. Am. Coll. Cardiol.* **49,** 2419–2426 (2007).

67. Arad, M. *et al.* Gene mutations in apical hypertrophic cardiomyopathy. *Circulation* **112,** 2805–2811 (2005).

68. Kronert, W. A., Melkani, G. C., Melkani, A. & Bernstein, S. I. A Failure to Communicate: MYOSIN RESIDUES INVOLVED IN HYPERTROPHIC CARDIOMYOPATHY AFFECT INTER-DOMAIN INTERACTION. *J. Biol. Chem.* **290,** 29270–29280 (2015).

69. Anan, R. *et al.* Prognostic implications of novel beta cardiac myosin heavy chain gene mutations that cause familial hypertrophic cardiomyopathy. *J. Clin. Invest.* **93,** 280–285 (1994).

70. Mora, R. *et al.* [Hypertrophic cardiomyopathy: infrequent mutation of the cardiac beta-myosin heavy-chain gene]. *Rev. Esp. Cardiol.* **59,** 846–849 (2006).

71. Otsuka, H. *et al.* Prevalence and distribution of sarcomeric gene mutations in Japanese patients with familial hypertrophic cardiomyopathy. *Circ. J.* **76,** 453–461 (2012).

72. Nishi, H., Kimura, A., Harada, H., Toshima, H. & Sasazuki, T. Novel missense mutation in cardiac beta myosin heavy chain gene found in a Japanese patient with hypertrophic cardiomyopathy. *Biochem. Biophys. Res. Commun.* **188,** 379–387 (1992).

73. Anderson, R. L. *et al.* Mavacamten stabilizes a folded-back sequestered super-relaxed state of β-cardiac myosin. Preprint at https://www.biorxiv.org/content/early/2018/02/16/266783 (2018).

74. Ho, C. Y. *et al.* Assessment of diastolic function with Doppler tissue imaging to predict genotype in preclinical hypertrophic cardiomyopathy. *Circulation* **105,** 2992–2997 (2002).

75. Andersen, P. S. *et al.* Adult-onset familial hypertrophic cardiomyopathy caused by a novel mutation, R694C, in the MYH7 gene. *Clinical genetics* **56,** 244–246 (1999).

76. Jaaskelainen, P. *et al.* The cardiac beta-myosin heavy chain gene is not the predominant gene for hypertrophic cardiomyopathy in the Finnish population. *J. Am. Coll. Cardiol.* **32,** 1709–1716 (1998).

77. Sakthivel, S., Joseph, P. K., Tharakan, J. M., Vosberg, H.-P. & Rajamanickam, C. A novel

missense mutation (R712L) adjacent to the ?active thiol? region of the cardiac ?-myosin heavy chain gene causing hypertrophic cardiomyopathy in an Indian family. *Hum. Mutat.* **15,** 298–299 (2000).

78. Fujita, H. *et al.* Characterization of mutant myosins of Dictyostelium discoideum equivalent to human familial hypertrophic cardiomyopathy mutants. Molecular force level of mutant myosins may have a prognostic implication. *J. Clin. Invest.* **99,** 1010–1015 (1997).

79. García-Giustiniani, D. *et al.* Phenotype and prognostic correlations of the converter region mutations affecting the β myosin heavy chain. *Heart* **101,** 1047–1053 (2015).

80. Consevage, M. W., Salada, G. C., Baylen, B. G., Ladda, R. L. & Rogan, P. K. A new missense mutation, Arg719Gln, in the beta-cardiac heavy chain myosin gene of patients with familial hypertrophic cardiomyopathy. *Hum. Mol. Genet.* **3,** 1025–1026 (1994).

81. Seebohm, B. *et al.* Cardiomyopathy mutations reveal variable region of myosin converter as major element of cross-bridge compliance. *Biophys. J.* **97,** 806–824 (2009).

82. Brenner, B., Seebohm, B., Tripathi, S., Montag, J. & Kraft, T. Familial hypertrophic cardiomyopathy: functional variance among individual cardiomyocytes as a trigger of FHC-phenotype development. *Front. Physiol.* **5,** 392 (2014).

83. Kawana, M., Sarkar, S. S., Sutton, S., Ruppel, K. M. & Spudich, J. A. Biophysical properties of human beta-cardiac myosin with converter mutations that cause hypertrophic cardiomyopathy. *Sci Adv* **3,** e1601959 (2017).

84. Enjuto, M. *et al.* Malignant Hypertrophic Cardiomyopathy Caused by the Arg723Gly Mutation in β -Myosin Heavy Chain Gene. *J. Mol. Cell. Cardiol.* **32,** 2307–2313 (2000).

85. Kraft, T. *et al.* Familial hypertrophic cardiomyopathy: Functional effects of myosin mutation R723G in cardiomyocytes. *J. Mol. Cell. Cardiol.* **57,** 13–22 (2013).

86. Blair, E., Price, S. J., Baty, C. J., Ostman-Smith, I. & Watkins, H. Mutations in cis can confound genotype-phenotype correlations in hypertrophic cardiomyopathy. *J. Med. Genet.* **38,** 385–8 (2001).

87. Davis, J. S. *et al.* The overall pattern of cardiac contraction depends on a spatial gradient of myosin regulatory light chain phosphorylation. *Cell* **107,** 631–641 (2001).

88. Moric, E. *et al.* Three novel mutations in exon 21 encoding beta-cardiac myosin heavy chain. *J. Appl. Genet.* **44,** 103–109 (2003).

89. Harada, H., Kimura, A., Nishi, H., Sasazuki, T. & Toshima, H. A missense mutation of cardiac beta-myosin heavy chain gene linked to familial hypertrophic cardiomyopathy in affected Japanese families. *Biochem. Biophys. Res. Commun.* **194,** 791–798 (1993).

90. Villard, E. *et al.* Mutation screening in dilated cardiomyopathy: prominent role of the beta myosin heavy chain gene. *Eur. Heart J.* **26,** 794–803 (2005).

91. Daehmlow, S. *et al.* Novel mutations in sarcomeric protein genes in dilated cardiomyopathy. *Biochem. Biophys. Res. Commun.* **298,** 116–20 (2002).

92. Liu, C., Kawana, M., Song, D., Ruppel, K. M. & Spudich, J. A. Controlling load-dependent kinetics of beta-cardiac myosin at the single-molecule level. *Nat. Struct. Mol. Biol.* **25,** 505–514 (2018)

93. Lin, T., Greenberg, M. J., Moore, J. R. & Ostap, E. M. A Hearing-Loss Associated Myo1c Mutation (R156W) Decreases the Myosin Duty Ratio and Force Sensitivity. *Biochemistry* **50,** 1831–1838 (2011).

94. Klaassen, S. *et al.* Mutations in sarcomere protein genes in left ventricular noncompaction. *Circulation* **117,** 2893–2901 (2008).

95. Kamisago, M. *et al.* Mutations in Sarcomere Protein Genes as a Cause of Dilated Cardiomyopathy. *N. Engl. J. Med.* **343,** 1688–1696 (2000).

96. Aksel, T., Choe Yu, E., Sutton, S., Ruppel, K. M. & Spudich, J. A. Ensemble force changes that result from human cardiac myosin mutations and a small-molecule effector. *Cell Rep.* **11,** 910–920 (2015).

97. Spudich, J. A. *et al.* Effects of hypertrophic and dilated cardiomyopathy mutations on power output by human β-cardiac myosin. *J. Exp. Biol.* **219,** 161–7 (2016).

98. Al-Khayat, H. A., Kensler, R. W., Squire, J. M., Marston, S. B. & Morris, E. P. Atomic model of the human cardiac muscle myosin filament. *Proc. Natl. Acad. Sci. U. S. A.* **110,** 318–23 (2013).
