# Supplementary material for: Hypertrophic cardiomyopathy disease results from disparate impairments of cardiac myosin function and auto-inhibition
Source: Nat Commun. 2018 Oct 1;9:4019. doi: 10.1038/s41467-018-06191-4 (PMC6167380; doi:10.1038/s41467-018-06191-4)
Supplement: Supplementary file 3 — Description of Additional Supplementary Files [file 41467_2018_6191_MOESM3_ESM.docx]

**Description of Additional Supplementary Files**

File Name: Supplementary Data 1

Description: Optimized quasi-atomic model of the sequestered state of bovine β-cardiac myosin.

File Name: Supplementary Data 2

Description: Optimized quasi-atomic model of the sequestered state of human β-cardiac myosin.

File Name: Supplementary Data 3

Description: Analysis of the structural and functional consequences of 178 HCM β-cardiac myosin mutations. Mutations have been classified in six groups. The color code in the row “impairment” depends on the class: black, motor function/stability mildly altered; blue: motor function altered- without predicted effect on the IHM; purple, protein stability and motor function altered; orange, sequestered state and PPS stability altered; yellow, sequestered state and motor function altered; green sequestered state destabilized. Extra references with color code in order to classify them depending on the experiments done in these cited papers: blue, studied from patient biopsies (β-cardiac myosin); orange, studied from recombinant/fibers from another organism. Also includes recombinant human myosin but other isoforms (for example alpha cardiac); red, studied with recombinant human β-cardiac myosin. NPI: not part of intra-molecular interface; BH: blocked head; FH: free head; PR: post-rigor; PPS: pre-powerstroke.

File Name: Supplementary Data 4

Description: Analysis of the structural and functional consequences of 23 DCM β-cardiac myosin mutations. Mutations have been classified in the six groups. The color code in the row “impairment” depends on the class: black, motor function/stability mildly altered; blue: motor function altered- without predicted effect on the IHM; purple, protein stability and motor function altered; orange, sequestered state and PPS stability altered; yellow, sequestered state and motor function altered; green sequestered state destabilized. References are cited with color code in order to indicate which experiments were done to study these mutations in these papers: blue, studied from patient biopsies (βcardiac myosin); orange, studied from recombinant/fibers from another organism or recombinant human myosin corresponding to other isoforms (for example alpha cardiac); red, studied with recombinant human β-cardiac myosin. NPI: not part of intra-molecular interface; BH: blocked head; FH: free head; PR: post-rigor; PPS: pre-powerstroke.
